# Impact of Sarcopenia and Serum Creatinine on Clinical Outcomes after Tace in Hepatocellular Carcinoma

**DOI:** 10.7150/jca.127063

**Published:** 2026-03-25

**Authors:** Anna Rossetto, Valli De Re, Luca Montaldo, Vittorio Bresadola, Aron Zompicchiatti, Massimo Sponza, Daniele Piccolo, Giorgio Soardo, Serena Battista, Alessandro Mangogna, Alessandro Uzzau

**Affiliations:** 1 Department of General Surgery, University of Udine, ASUFC, San Daniele del Friuli (UD), Italy.; 2 Department of Translational and Advanced Tumour Diagnostics, Immunopatologia e Biomarcatori Oncologici (IBO) Unit, Centro di Riferimento Oncologico di Aviano (CRO), IRCCS, Aviano, Italy.; 3 Radiology Department, University of Udine, ASUFC, San Daniele del Friuli (UD), Italy.; 4 Area Medica Department, General Surgery, University Hospital of Udine, ASUFC, Udine, Italy.; 5 Department of Radiology, Interventional Radiology Unit, University of Udine, ASUFC, Udine, Italy.; 6 Department of Clinical, Surgical, Diagnostic and Pediatric Sciences, University of Pavia, Pavia, Italy.; 7 Unit of Neurosurgery, Department of Head-Neck and Neuroscience, ASUFC, Udine, Italy.; 8 Area Medica Department, University of Udine, Liver Unit, ASUFC, Udine, Italy.; 9 Pathology Department, University of Udine, ASUFC, Udine, Italy.; 10 Department of Medicine (DMED), University of Udine, Institute of Pathology, ASUFC, Udine, Italy.

**Keywords:** Hepatocellular carcinoma, Sarcopenia, Inflammation, Chemoembolization, Disease-free survival, Tumor response

## Abstract

**Background:**

Transarterial chemoembolization (TACE) is the standard treatment for intermediate unresectable hepatocellular carcinoma (HCC); however, reliable prognostic markers are still lacking. Sarcopenia has been proposed as a negative prognostic factor in HCC, but its impact on TACE outcomes remains unclear.

**Methods:**

We retrospectively analyzed 48 HCC patients treated with TACE or transarterial embolization (TAE) at our institution (between 2013 and 2020). Sarcopenia was assessed on computed tomography (CT) or magnetic resonance imaging (MRI) scans at baseline, one month, and six months after treatment according to RECIST criteria.

**Results:**

At six months, 27 patients (61.4%) achieved complete or partial response, while 17 (38.6%) experienced stable or progressive disease; four patients were excluded due to missing follow-up data. Sarcopenia was more frequent among responders, increasing from 13.5% at baseline to 22.2% in 6 months, while it was initially absent in non-responders. Conversely, non-responders showed a later increase in sarcopenia (0% at baseline to 29.6% at 6 months), suggesting that late sarcopenia might reflect treatment-related metabolic changes. Overall, the prevalence of sarcopenia increased during follow-up. New-onset sarcopenia was more frequent in non-responders and was associated with lower serum creatinine levels , suggesting a possible link between treatment-related muscle loss and poor therapeutic response. Kaplan-Meier showed that smoking status was associated (p = 0.01) with poorer response at 6 months (), while sarcopenia and low creatinine levels showed borderline associations (p = 0.095).

**Conclusion:**

In this exploratory study, baseline CT-defined sarcopenia was not significantly associated with short-term response to TACE. However, treatment-related sarcopenia with low creatinine levels may reflect frailty during follow-up, and poorer therapeutic response. Given the small sample size and limited number of sarcopenic patients, these findings should be considered hypothesis-generating and require validation in larger prospective studies.

## Introduction

Hepatocellular carcinoma (HCC) remains a major clinical challenge, ranking as the third leading cause of cancer-related death and the sixth most common cancer worldwide 1. The introduction of hepatitis B virus (HBV) vaccination program and the development of direct-acting antivirals against the hepatitis C virus (HCV) have substantially reduced the incidence of virus-related HCC. Consequently, metabolic dysfunction-associated liver disease (MALFD) has emerged as an increasingly relevant etiological factor, both in cirrhotic and non-cirrhotic HCC patients 2.

According to the revised 2018 European Association for the study of the liver (EASL) Barcelona Clinic Liver Cancer (BCLC) guidelines, transarterial chemoembolization (TACE) represents the standard of care for patients with intermediate-stage HCC (BCLC B) 3,4. TACE combines intra-arterial delivery of cytotoxic agents with arterial embolization, leading to tumor ischemia and necrosis. Since HCC is highly chemoresistant, transarterial embolization (TAE) alone may achieve comparable outcomes to TACE. Moreover, beyond BCLC stage B, carefully selected patients with stage A or C disease may also be considered for TACE within a multidisciplinary setting 5. Meanwhile, systemic treatment options for HCC have expanded to include multikinase inhibitors (MKIs), anti-VEGF agents, immune checkpoint inhibitors (ICIs), and combination regimens 6-8.

Despite these advances, prognosis after TACE remains heterogeneous, highlighting the need for reliable biomarkers to refine patient stratification beyond traditional staging systems. Sarcopenia, defined as the progressive loss of skeletal muscle mass and function, and systemic inflammatory indices reflecting immune imbalance have gained increasing attention as prognostic markers in cancer, including HCC 9-12.

Sarcopenia may be primary (age-related), or secondary (disease-related), and its adverse prognostic impact has been well established in HCC patients undergoing liver resection or transplantation 10,13. However, evidence regarding its prognostic significance in patients undergoing TACE remains limited and requires further validation 1,9,13-17. Similarly, inflammatory biomarkers have shown promise in improving prognostic stratification, potentially enabling more personalized treatment strategies 18,19.

In this context, the present retrospective monocentric study aimed to investigate whether sarcopenia and inflammatory biomarkers are associated with treatment response and recurrence after TACE in HCC patients, in comparison with conventional prognostic factors.

## Materials and Methods

### Study design and patient selection

This retrospective observational study included 48 patients with HCC treated with TACE or TAE at the University Hospital of Udine between July 2013 and January 2020.

Inclusion criteria were age ≥ 18 years, radiological or histological diagnosis of HCC, and no previous treatment for HCC.

Patients were excluded if they had undergone previous hepatic surgery or ablation, lacked baseline or follow-up (T1, 1 month; T2, 6 months) imaging with computed tomography (CT) or magnetic resonance imaging (MRI), or if sarcopenia assessment at the L3 vertebral level was not feasible due to incomplete imaging or artifacts.

### Transarterial procedures

The indication for TACE with doxorubicin or for TAE was established by a multidisciplinary tumor board. All procedures were performed according to institutional standards, most commonly using ethanol-lipiodol embolization 20

### Outcome assessment

Tumor response was evaluated at T1 and T2 after treatment using the Response Evaluation Criteria in Solid Tumors (RECIST) on dynamic CT or MRI scans 21. Responders were defined as patients achieving complete response (CR: disappearance of any intra-tumoral arterial enhancement) or partial response (PR: ≥ 30% reduction in the sum of target lesion diameters); while non-responders include those with stable (SD) or progressive disease (PD).

Disease-free survival (DFS) was calculated from baseline (T0) to SD or PD within six months.

#### Sarcopenia assessment

Skeletal muscle mass was quantified on axial CT or MRI images at the L3 vertebral level at baseline (T0), at 1 month (T1), and six months (T2). Psoas muscle diameters were used to calculate the skeletal muscle index (SMI), defined as the sum both diameters, and the psoas muscle index (PMI), normalized for height² (mm/m^2^).

Sarcopenia was defined according to previously published sex-specific cut-offs (PMI < 42.28 mm/m² for men and < 37.42 mm/m² for women) 22.

#### Clinical and laboratory data

Baseline clinical variables included age, sex, smoking status, alcohol consumption, body mass index, diabetes, and viral etiology.

Patients were classified as never drinkers or current drinkers; ex-drinkers were those who had abstained from alcohol for at least six months prior to enrollment. HCC was classified as viral-related when hepatitis B surface antigen or anti-HCV antibodies were present, or when patients had history of antiviral therapy for HCV, Body mass index (BMI) was categorized as overweight (> 24.9 kg/m^2^) or non-overweight (≤ 24.9 kg/m^2^) according to WHO criteria 23. Diabetes mellitus was defined as fasting glucose ≥ 110mg/dL.

Liver function was assessed using serum albumin, bilirubin, alfa-fetoprotein (AFP), creatinine, international normalized ratio (INR) clotting time, Child-Pugh class, and MELD scores 24.

Inflammatory indices at baseline T0 included: neutrophil-to-lymphocyte ratio (NLR, cut-off = 2.4), platelet-to-lymphocyte ratio (PLR, cutoff = 150), and lymphocyte-to-C-reactive protein ratio (LCRR, cutoff = 6000). Tumor related variables included tumor size (≤ 20 mm, 20-57 mm, > 57 mm), number of nodules, presence of portal vein thrombosis, and BCLC stages (0, A, B, C, D) 25.

### Statistical analysis

Continuous variables were expressed as median (interquantile range, IQR) and categorical variables as percentages. Baseline differences between groups were analyzed using non-parametric ANOVA. Group comparisons were performed using appropriate non-parametric tests. Differences in categorical variables were analyzed with Fisher's exact test. Variable with p < 0.01 in univariate analysis were entered into multivariate logistic regression model to identify predictors of sarcopenia and treatment response. Disease-free survival (DFS) analyses were conducted using Kaplan-Meier curves, and receiver operating characteristic (ROC) curves were used to-determine optimal Youden cutoff for continuous predictors.

Statistical analyses were performed using R and MedCalc software.

## Results

### Patient characteristics

A total of 48 patients with unresectable HCC treated with TACE or TAE at the University Hospital of Udine between July 2013 and January 2020 were included in the analysis. Baseline characteristics are summarized in** Table 1**. The median age was 67.3 years, with a predominance of males. Diabetes was present in 26.8% of cases and 52.7% of patients were current or former smokers. Most patients had preserved liver function (Child-Pugh A; 69.2%), while 9.7% presented with partial portal vein thrombosis. According to the BCLC classification, 29.5% of patients were stage 0, 31.8% stage A and 38.6% stage B.

### Predictors of complete response and treatment outcome

At one-month post-treatment, a complete response (CR) was achieved in 60.4% of patients, decreasing to 43.2% at six months. Stable disease (SD) and a progressive disease (PD) were observed in 4.5% and 34.1% of patients, respectively (**Table 2**).

Smoking history (current or former) was significantly associated with non-CR status at both 1 and 6 months post-treatment. Additionally, a non-exotoxic etiology (non-viral/non-alcoholic) was linked to a higher proportion of non-CR outcomes at 6 months (**Table 3A**).

A higher spoas muscle index (PMI) and serum albumin levels were both associated with CR at 6 months (**Table 3B**).

When comparing responders (CR + PR) and non-responders (SD + PD) at 1 and 6 months, no statistically significant associations were identified. However, several variables showed a trend toward significance (p < 0.15) including the presence of multiple nodules (≥ 1), smoking status, portal vein thrombosis, BCLC stage, patient age, AFP and creatinine levels (**Tables 3A and 3B**).

Comprehensive data for all variables are available in **Supplementary Tables 1, 2 and 3.**

### Sarcopenia and prognosis

The association between sarcopenia and treatment response is summarized in **Table 2**. During the 6-month follow-up, the prevalence of sarcopenia increased from 12.5% at baseline to 27.3%.

Among patients with complete response, sarcopenia prevalence rose 17.2% at 1 month to 26.3% at 6 months, whereas it remained largely unchanged in the other response categories.

Among non-responders at 6 months, all six patients with baseline sarcopenia (five with CR and one without) remained sarcopenic, while six additional cases were identified (**Table 3A**). Of these new cases, four patients (66.7%) developed progressive disease, while two achieved a complete or partial response.

Among the 32 patients without baseline sarcopenia, 13 (41%) developed PD. However, the difference in PD rate between patients with and without sarcopenia, did not reach statistical significance (4/6 vs 13/32, Fisher's exact test p = 0.38). Notably, SD or PD disease occurred only in patients who developed new-sarcopenia, suggesting a possible link between treatment-related muscle loss and poorer therapeutic response.

**Figure 1:**
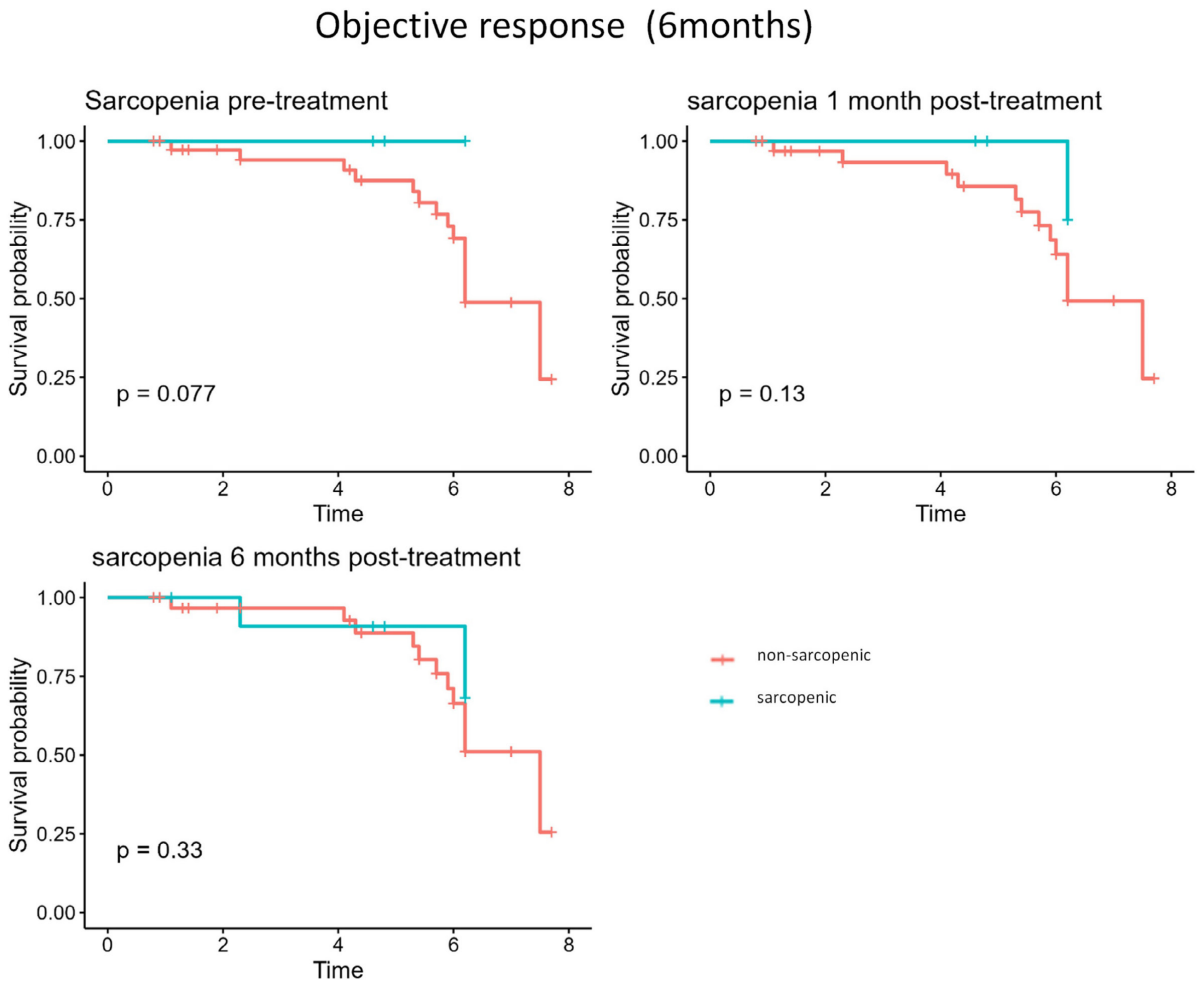
Kaplan-Meier survival curves comparing sarcopenic and non-sarcopenic patients at baseline, 1 month, and 6 months after treatment for treatment response (CR +PR vs SD + PD).

Kaplan-Meier curves (**Figure 1**) showed a trend towards poorer 6-month objective response in sarcopenic patients compared to non-sarcopenic patients. At baseline, sarcopenic patients had a lower survival probability (p = 0.077), while differences at 1 month (p = 0.13) and 6 months (p = 0.33) were not significant.

Overall, although sarcopenia prevalence progressively increased (from 12.2% to 26.7%), the proportion of PD remained comparable between groups.

### Sarcopenia and creatinine

**Figure 2:**
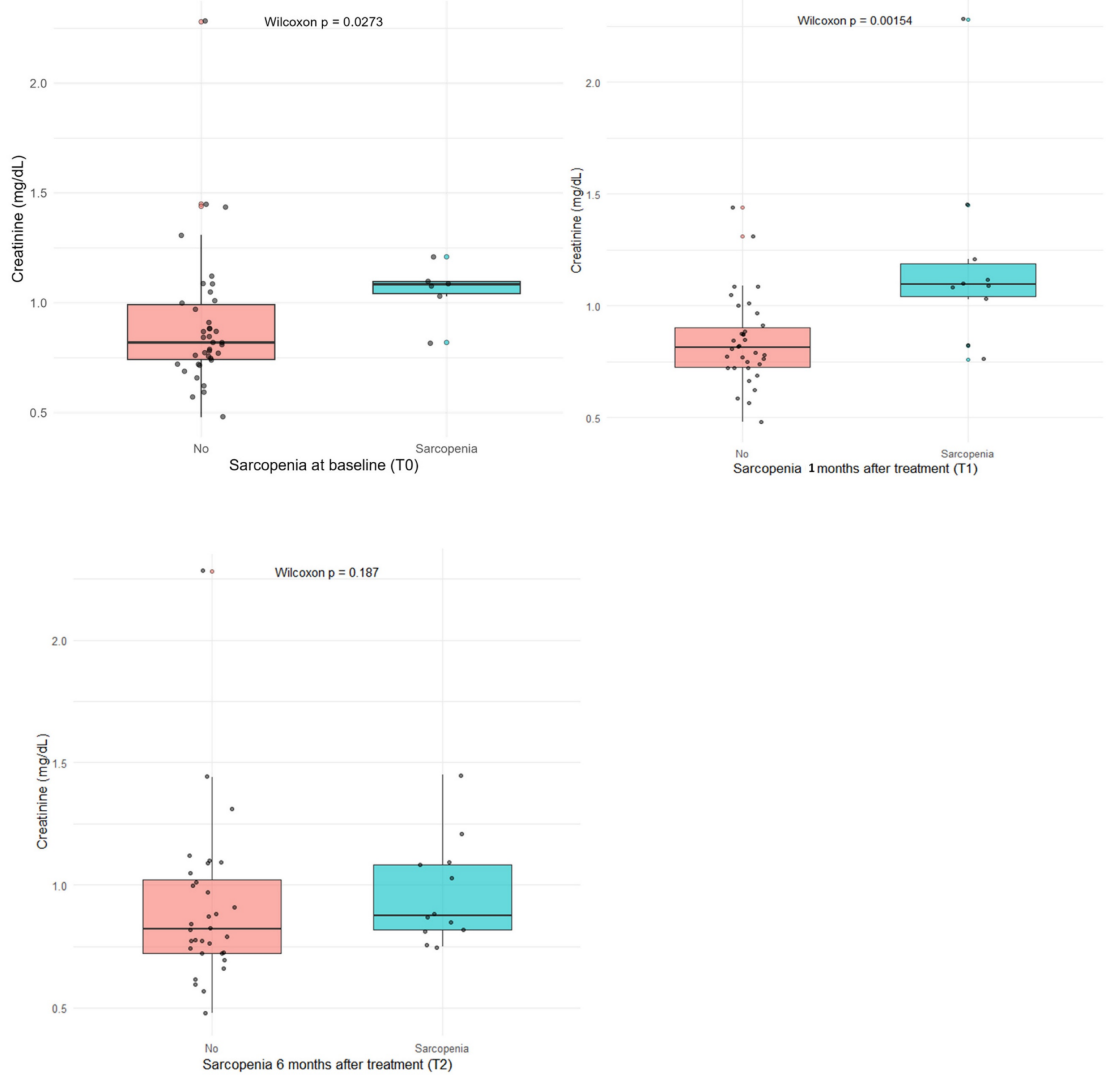
Wilcoxon test comparing of serum creatinine levels (mg/dL) between sarcopenic and non-sarcopenic patients at baseline, 1 month, and 6 months after treatment.

At logistic regression, baseline sarcopenia showed a borderline association with serum creatinine levels (p = 0.06), whereas a significant association emerged at 1 month post-treatment (p = 0.006). Wilcoxon tests confirmed higher creatinine levels in sarcopenic patients both at baseline (p = 0.027) and, more markedly, at 1 month (p = 0.002) (**Figure 2**).

Receiver operating characteristic (ROC) analysis identified an optimal serum creatinine cut-off of >1.0 mg/dL to discriminate patients with sarcopenia.

Among sarcopenic patients, non-responders tended to have lower creatinine levels (median 0.79 vs. 1.10 mg/dL, p = 0.06), although the small sample size limits statistical power (**Table 4**).

Kaplan-Meier analysis showed better outcomes in patients with baseline creatinine > 1.0 mg/dL (p = 0.035), consistent with the trend observed for sarcopenia (p = 0.077, **Figure 1**).

## Discussion

HCC develops through multiple mechanisms involving alterations to the tumor microenvironment and systemic metabolic dysfunction 8. As obesity and type 2 diabetes become more prevalent, metabolic dysfunction-associated fatty liver disease (MAFLD) is emerging as a leading cause of HCC, gradually replacing viral hepatitis as the predominant cause 26. This epidemiological shift highlights the importance of metabolic and inflammatory factors in the pathogenesis and prognosis of HCC.

Sarcopenia has emerged as an adverse prognostic factor in chronic liver disease and various cancers 27,28. Biologically, muscle depletion compromises protein reserves, impairs tissue repair, and disrupts immune-inflammatory balance, ultimately worsening survival. Most previous studies of HCC have focused on patients undergoing curative treatments such as resection or transplantation. The role of sarcopenia in patients receiving TACE or TAE remains poorly defined 1,9,10,13-17. This study explores the prognostic role of sarcopenia and creatinine dynamics in HCC patients undergoing TACE.

We observed a progressive increase in the prevalence of sarcopenia during the follow-up period. Although sarcopenia at baseline was not significantly associated with treatment response, patients who developed new-onset sarcopenia, particularly in association with lower serum creatinine levels (< 1 mg/dL), tended to experience poorer outcomes. However, given the limited number of events and borderline statistical significance, these findings should interpret with caution and primarily as exploratory signals rather than definitive prognostic indicators. This observation aligns with previous evidence indicating that creatinine levels reflect skeletal muscle mass loss in patients with chronic liver disease 29,30. As creatine is synthesized in the liver and stored in skeletal muscle, where it is metabolized to creatinine, both hepatic dysfunction and muscle depletion can both reduce serum creatinine levels. Interestingly, in our cohort, baseline sarcopenia was more frequently observed in patients with higher creatinine levels (> 1 mg/dL). These patients also tended to show a more favorable short-term response. One possible explanation is that elevated creatinine levels in these patients may indicate residual confounding factors, preserved renal function or higher metabolic activity related to liver regeneration. This was not fully captured in the retrospective analysis. Conversely, lower creatinine levels may be representing a surrogate of frailty or reduced muscle reserve, which is consistent with poorer outcomes observed in this subgroup. These findings required validation in larger cohorts.

Several imaging-based methods have been developed to assess sarcopenia 15,31-33. We used the psoas muscle index (PMI), which is calculated from simple linear diameters at the L3 vertebral level, and normalized for the patient's height. Although, less comprehensive than total skeletal muscle area (SMI), the PMI is simple and reproducible, and correlates with global muscle status 9,22,32. Future studies should confirm whether the PMI adequately captures systemic muscle depletion in comparison with volumetric or radiomic methods.

The pathogenesis of sarcopenia in liver disease is multifactorial, involving both hepatopathy- and tumor- related mechanisms 34. Chronic inflammation can lead to oxidative stress and the release of cytokines, which can impair protein synthesis and increase myostatin expression that inhibits muscle mass 1,7,14,32,33. Moreover, changes in body composition, including loss of muscle and visceral adiposity, further complicate prognostic interpretation. For example, body mass index (BMI) can be misleading in HCC, acting as either a protective or an adverse factor depending on the disease context.

Our study has several limitations. The retrospective design, small sample size, and relatively short follow-up period restrict statistical power and causal inference. We also did not account for potential confounders such as nutritional status, hormonal influences, or gut microbiota composition. Nevertheless, our findings suggest that creatinine and sarcopenia dynamics may be relevant for predicting outcomes in patients undergoing TACE.

In the era of precision oncology, integrating body composition metrics, molecular biomarkers, and radiomic or artificial intelligence-based tools could improve patient classification and guide personalized treatment. Nutritional and physical interventions, such as branched-chain amino acid supplementation, and lifestyle modifications, may also help to preserve muscle mass and improve outcomes 35-39.

## Conclusion

Although sarcopenia is widely recognized as an adverse prognostic factor in cancer, CT-based sarcopenia was not directly associated with the short-term response to TACE in this cohort of HCC patients. Instead, sarcopenia combined with low serum creatinine levels (≤ 1.0 mg/dL) emerged as a potential marker of frailty and poor therapeutic response, possibly reflecting early metabolic and muscle changes resulting from treatment or disease progression. Overall, the absence of robust statistical significance, together with the small number of sarcopenic patients, suggests that sarcopenia and creatinine levels should be considered dynamic biomarkers that merit further investigation, before they can be incorporated into clinical decision-making.

## Supplementary Material

Supplementary tables.

## Figures and Tables

**Figure 3:**
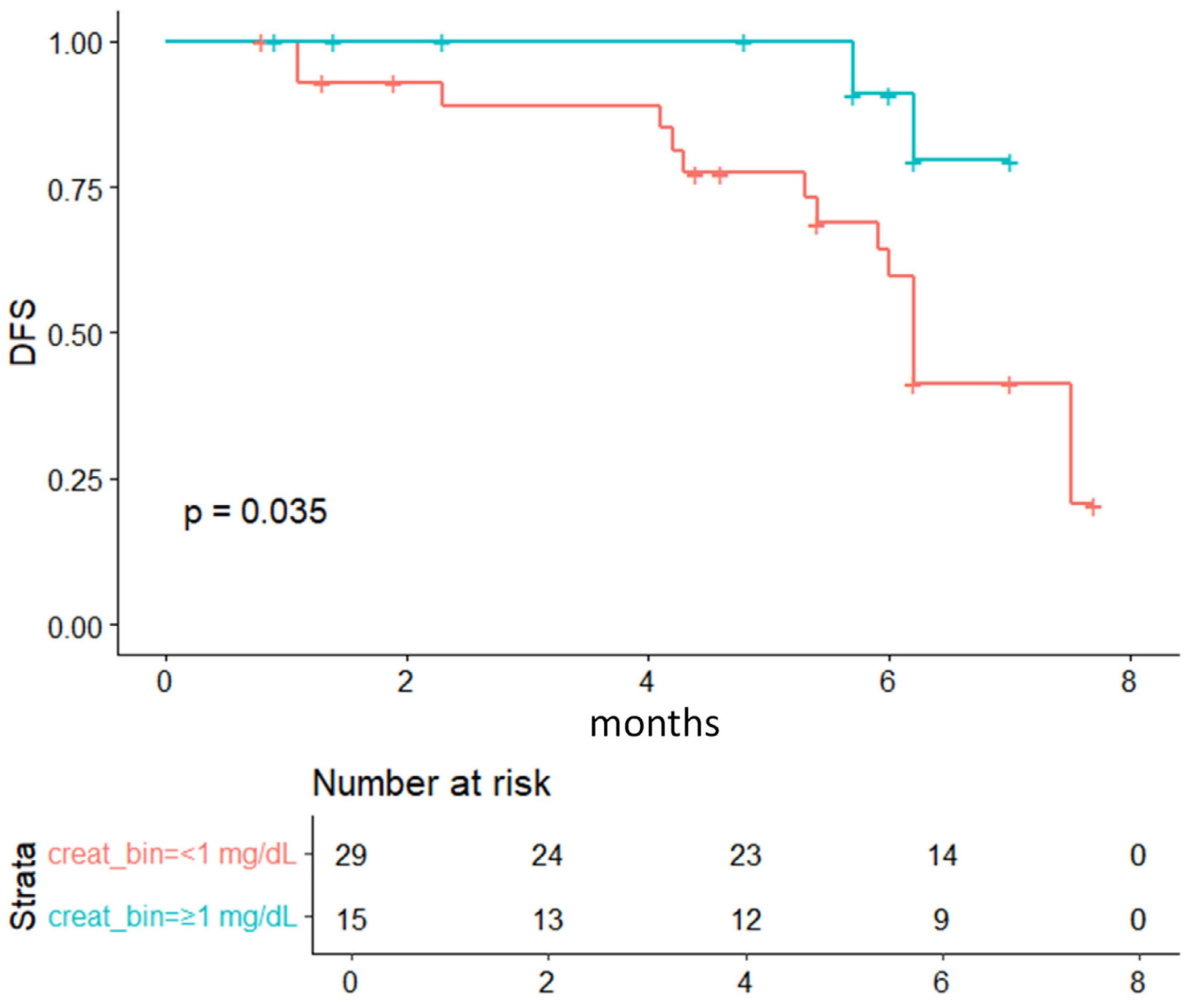
Kaplan-Meier survival curves comparing patients stratified by serum creatine levels (<1 mg/dL vs ≥1 mg/dL) according to treatment response (CR +PR vs SD + PD).

**Table 1:** Baseline characteristics of the study cohort

**Demographic** **characteristic**		**n**
Male sex, N (%)	36 (75.0%)	48
Age (years)(mean, range)	67.3 (±8.06)	48
**Risk factors for HCC**		
Overweight (BMI kg/m^2^)	24 (50.0%)	48
Diabetes mellitus (≥100mg/dL)	11 (26.8%)	41
Hepatitis B and/or Hepatitis C	20 (40.8%)	41
Non-exotoxic etiology	26 (63.4%)	41
Alcohol consumption^#^	28 (70.0%)	40
Smoker^#^ (≥25 cigarette/day)	20 (52.6%)	38
**Blood laboratory data**		
Alfa-fetoprotein (ng/mL)	7.9 (4.7-16.6)	48
Hemoglobin (g/dL)	13.4 (±1.97)	48
Coagulation (International Normalized Ratio)	1.2 (1.1-1.2)	48
Total bilirubin (mg/dL)	1.0 (0.9-1.6)	48
Creatinine (mg/dL)	0.8 (0.8-1.02)	48
Albumin (g/L)	39.3 (36.6-42)	42
C reactive protein (mg/dL)	2.8 (0.87-4-9)	44
White blood cells count (cells/µL)	4.6 (±1.89)	48
Neutrophils count (cells/µL)	2.7 (2.0-3.3)	48
Lymphocytes count (cells/µL)	1.1 (0.85-1.6)	48
Platelets median (x10^3^/µL)	77.0 (58.0-106)	47
NLR	2.2 [1.68-3.12]	48
PLR	76.8 (66.6-95.2)	48
LCRR	5320.0 [2702.0-11230.0]	43
**HCC prognostic factors and classification**		
Child-Pugh A	34 (69.2%)	39
MELD score A C D	27 (56.2%) 10 (20.8%) 11 (22.9%)	48 48 48
BCLC staging		
0	13 (29.5%)	44
A	14 (31.8%)	44
B	17 (38.6%)	44
Focal nodular HCC (one nodule)	19 (46.3%)	41
Tumor size (cm) , na = 2		
≤ 2	14 (31.8%)	44
≥ 6	10 (22.7%)	44
Portal vein thrombosis	4 (9.7%)	41

Continuous variables are summarized as arithmetic mean ± SD (95% CI) when approximately normal (Shapiro-Wilk p≥0.05), otherwise as median (IQR); categorical variables as number (percentage). n, total cases tested, they may differ across variables and between columns due to missing data. ^#^, includes both current and former users. NLR, ratios between absolute neutrophils count and absolute lymphocytes count; PLR, platelets and lymphocytes; LCRR, lymphocytes and C-reactive protein ratio.

**Table 2:** Sarcopenia and treatment response over time

**Sarcopenia**		
**metric**	**Value (median, IQR or n, %)**	
Psoas muscle index (PMI cm^2^/m^2^), n=48		
Before treatment	4.95 (4.42-5.27)	
1 month later	4.72 (4.19-5.00)	
6 months later	4.53 (4.13-4.84)	
Sarcopenia at baseline (n=48)	6 (12.5%)	
Sarcopenia 1 month (n=48)	10 (20.8%)	
Sarcopenia at 6 months, (n=44)	12 (27.3%)	
**Response Over Time**		
**Timepoint**	**Response type**	**n/tot, (%)**
First response (1 month) (n=48)		
	CR	29 (60.4%)
	PR	15 (31.2%)
	SD	0
	PD	4 (8.3%)
Next response (6th month) (n=44)		
	CR	19 (43.2%)
	PR	8 (18.2%)
	SD	2 (4.5%)
	PD	15 (34.1%)

Note: The number of patients with available data (n) may vary across variables and between columns due to missing data. CR=complete response, PR=partial response, SD=stable disease, PD=progressive disease

**Table 3A:** Relevant clinical and categorical variables for both complete response (CR) vs non-complete response (non-CR) (**A**) and overall responders (CR+PR) vs non-responders (SD+PD) (**B**) at 1 and 6 months.

**A.**							
**Variable**	**CR (1 mo) n/N (%)**	**non-CR (1 mo) n/N (%)**	**p**		**CR (6 mo) n/N (%)**	**non-CR (6 mo) n/N (%)**	**p**
Smokers or ex smokers	9/25 (36.0%)	10/12 (83.3%)	0.01		6/19 (31.6%)	14/19 (73.7%)	0.02
Non-exotoxic etiology^ #^	14/25 (53.8%)	12 /14 (85.7%)	0.08		9/20 (45.0%)	17/21 (81.0%)	0.02
Sarcopenia T0	5/29 (17.2%)	1/19 (5.3%)	0.38		5/19 (26.3%)	1/25 (4.0%)	0.07
Sarcopenia T1	9/29 (31.0%)	1/19 (5.3%)	0.06		7/19 (36.8%)	3/25 (12.0%)	0.07
Sarcopenia T2	--	--	--		7/19 (36.8%)	5/25 (20.0%)	0.31
**B.**							
**Variable**	**CR+PR (1 mo) n/N (%)**	**SD+PD (1 mo) n/N (%)**	**p**	**CR+PR (6 mo) n/N (%)**		**SD+PD (6 mo) n/N (%)**	**p**
Focal nodular HCC (one nodule)	16/38 (42.1%)	3/3 (33.3%)	0.09	13/25 (52.0%)		10/16 (62.5%)	0.52
Smokers or ex smokers	19/37 (51.4.5%)	1/1 (100.0%)	1.00	10/24 (41.7%)		10/14 (71.4%)	0.10
Portal vein thrombosis	4/38 (10.5%)	0/3 (0.0%)	1.00	4/25 (16.0%)		0/16 (0.0%)	0.14
BCLC stage 0A vs B	26/41 (36.6%)	1/3 (33.3%)	0.55	20/28 (71.4%)		7/16 (56.3%)	0.11
Sarcopenia T0	6/44 (13.6%)	0/4 (--)	1.00	6/27 (22.2%)		0/17 (--)	0.07
Sarcopenia T1	10/44 (22.7%)	0/4 (--)	0.57	8/27 (29.6%)		2/17 (11.8%)	0.27
Sarcopenia T2	--	--	--	8/27 (29.6%)		4/17 (29.6%)	0.74
							

Data are expressed as number of positive cases/total evaluable patients (%).The number of patients with available data may differ across variables due to missing data. Variables with p < 0.10 and clinically relevant variables (e.g., BCLC stage, portal vein thrombosis, sarcopenia overtime) are shown and retained for further analysis. #, metabolic or alcohol consumption. P values were calculated using Fisher's exact test.

**Table 3B:** Comparison of relevant biochemical and continuous variables for both complete response (CR) vs non-complete response (non-CR) (**A**) and overall responders (CR+PR) vs non-responders (SD+PD) (**B**) over time.

**A.**										
**Variable**	**CR (1 mo) n/N median [IQR]**		**Non-CR (1 mo) n/N median [IQR]**		**P**	**CR (6 mo) n/N median [IQR]**		**Non-CR (6 mo) n/N median [IQR]**		**P**
Albumin (g/L)	24/41	39.30 [37.17-42.00]	17/41	37.90 [36.10-41.00]	0.53	16/38	39.35 [38.22-42.80]	22/38	37.70 [35.20-40.85]	0.05
Creatinine (mg/dL)	29/47	0.84 [0.72-1.08]	18/47	0.83 [0.77-0.96]	0.99	19/44	1.00 [0.79-1.15]	25/44	0.81 [0.75-0.91]	0.06
C reactive protein (mg/dL)	25/43	1.59 [0.63-3.90]	18/43	3.34 [1.22-5.08]	0.19	18/39	0.98 [0.62-3.14]	21/39	3.05 [1.25-4.92]	0.07
PMI_pre	29/48	46.02 [43.56-52.20]	19/48	49.95 [48.96-52.48]	0.14	19/44	45.68 [41.96-50.77]	25/44	49.63 [45.88-54.33]	0.03
PMI_post	29/48	44.64 [40.30-48.91]	19/48	48.46 [46.85-50.40]	0.03	19/44	43.13 [39.60-47.70]	25/44	48.46 [46.02-51.94]	0.01
**B.**										
**Variable**	**Responders (CR+PR, 1 mo)** **n/N median [IQR]**		**Non-Responders (SDR+PD, 1 mo)** **n/N median [IQR]**		**p**	**Responders (CR+PR, 6 mo)** **n/N median [IQR]**		**Non-Responders (SDR+PD, 6 mo)** **n/N median [IQR]**		**p**
Age, years	44/48	65.02 [61.45-73.19]	4/48	73.42 [70.74-76.73]	0.07	27/44	65.43 [61.34-73.87]	17/44	65.06 [62.69-73.72]	0.89
Alfa-feto protein (ng/ml)	44/47	7.80 [4.73-14.15]	3/47	10.80 [7.25-15.00]	0.79	27/44	6.20 [3.90-9.40]	17/44	9.90 [6.20-23.60]	0.06
Creatinine (mg/dL)	44/47	0.84 [0.75-1.06]	3/47	0.81 [0.79-0.86]	0.78	27/44	0.88 [0.76-1.09]	17/44	0.79 [0.76-0.87]	0.10

Data are expressed as median [IQR]. Variables with p < 0.15 in at least one comparison are shown. Variables with p < 0.10-0.15 and those considered clinically relevant were included in the logistic regression analysis for sarcopenia P₁ values were calculated using the Mann-Whitney U test.

**Table 4:** Creatinine levels in sarcopenic (6mo) and non-sarcopenic patients

			Creatinine (mg/dL)		
			median	IQR	p
without sarcopenia		responder n = 19	0.84	(0.72-1.01)	0.56
		non-responder n = 12	0.79	(0.72-0.88)	
sarcopenic (6mo)		responder n = 7	1.1	(1.06-1.33)	0.06
		non-responder n=5	0.79	(0.76-0.97)	
