# Supplementary material for: Impact of Sarcopenia and Serum Creatinine on Clinical Outcomes after Tace in Hepatocellular Carcinoma
Source: J Cancer. 2026 Mar 25;17(4):712–20. doi: 10.7150/jca.127063 (PMC13104724; doi:10.7150/jca.127063)
Supplement: Supplementary file 1 — Supplementary tables. [file jcav17p0712s1.pdf]

Supplementary Table 1. Comparison of variables to TACE treatment at 1 month and at 6 months

**Variables**	**First-response (1month)**		***P***	**Variables**	**Next response (6 months)**		***P***
**Overweight**, N (%)				Overweight, N (%)			
CR (n=32)	16	50.0%	0.92	CR (n=20)	8	40.0%	0.73
PR (n=16)	9	56.2%		PR (n=9)	5	55.6%	
SD (n=0)	-	-		SD (n=2)	1	50.0%	
PD (n=4)	2	50%		PD (n=16)	10	62.5%	
**Hepatitis B, Hepatitis C**, N (%) na=8				Hepatitis B , Hepatitis C, N (%) na=7			
CR (n=29)	16	55.2%	0.61	CR (n=18)	10	55.6%	0.49
PR (n=12)	5	41.7%		PR (n=6)	3	50.0%	
SD (n=0)	-	-		SD (n=2)	2	100.0%	
PD (n=3)	1	33.3%		PD (n=14)	5	35.7%	
**Alcohol consumption** na=8				Alcohol consumption na=7			
CR (n=29)	11	37.9%	0.08	CR (n=18)	8	44.4%	0.36
PR (n=12)	9	75.0%		PR (n=6)	5	83.3%	
SD (n=0)	-	-		SD (n=2)	1	50.0%	
PD (n=3)	2	86.7%		PD (n=14)	6	42.9%	
**Alfa-feto protein** (ng/ml), mean±SD na=1				Alfa-feto protein (ng/ml), mean±SD			
CR (n=32)	954	±SD5157	0.74	CR (n=20)	28	±SD89	0.34
PR (n=16)	14	±SD12		PR (n=9)	3252	±SD9734	
SD (n=0)	-	-		SD (n=2)	8	±SD3	
PD (n=3)	11	±SD7.7		PD (n=16)	50	±SD99	
**Hemoglobin** (g/dL) ,mean±SD na=1				Hemoglobin (g/dL)			
CR (n=32)	13.3	±SD2.1	0.31	CR (n=20)	13.7	±SD2.2	0.78
PR (n=16)	13.9	±SD1.6		PR (n=9)	13.0	±SD2.0	
SD (n=0)	-	-		SD (n=2)	14.0	±SD0.4	
PD (n=3)	14.2	±SD1.1		PD (n=16)	13.8	±SD1.6	
**Coagulation** (INR index) na=5				Coagulation (INR index) n=4			
CR (n=29)	1.2	±SD0.3	0.54	CR (n=19)	1.2	±SD0.3	0.99
PR (n=15)	1.2	±SD0.2		PR (n=8)	1.2	±SD0.1	
SD (n=0)	-	-		SD (n=2)	1.2	±SD0.3	
PD (n=3)	1.1	±SD0.1		PD (n=15)	1.2	±SD0.2	
**Total bilirubin** (mg/dL) ,mean±SD na=5				Toal bilirubin (mg/dL) na=4			
CR (n=29)	1.17	±SD0.6	0.30	CR (n=19)	1.21	±SD0.8	0.51
PR (n=15)	1.52	±SD0.9		PR (n=8)	1.19	±SD0.6	
SD (n=0)	-	-		SD (n=2)	1.08	±SD0.8	
PD (n=3)	1.51	±SD0.5		PD (n=15)	1.57	±SD0.8	

**Albumin** (g/L), mean±SD na=7				Albumin (g/L), mean±SD na=6			
CR (n=27)	38.2	±SD6.1	0.97	CR (n=17)	40.7	±SD3.1	0.23
PR (n=16)	38.1	±SD4.1		PR (n=9)	37.6	±SD3.9	
SD (n=0)	-	-		SD (n=2)	38.7	±SD1.1	
PD (n=2)	39.1	±SD4.2		PD (n=13)	37.8	±SD5.6	
**C reactive protein** (mg/dL), mean±SD na=9				C reactive protein (mg/dL), mean±SD na=7			
CR (n=25)	5.4	±SD9.5	0.31	CR (n=18)	4.4	±SD9.8	0.91
PR (n=14)	3.4	±SD2.4		PR (n=7)	3.6	±SD2.0	
SD (n=0)	-	-		SD (n=2)	0.6	±SD0.5	
PD (n=4)	11.0	±SD15.3		PD (n=13)	4.5	±SD5.2	
**White blood cell** count (cells/µL), mean±SD na=8				White blood cell count (cells/µL), mean±SD na=4			
CR (n=29)	4.7	±SD1.9	0.64	CR (n=20)	4.8	±SD2.0	0.36
PR (n=15)	5.2	±SD2.0		PR (n=9)	4.7	±SD1.5	
SD (n=0)	-	-		SD (n=2)	7.4	±SD3.9	
PD (n=3)	4.1	±SD0.8		PD (n=16)	4.8	±SD1.9	
**Neutrophils** count (cells/µL), mean±SD na=5				Neutrophils count (cells/µL), mean±SD na=4			
CR (n=29)	2.8	±SD1.3	0.62	CR (n=20)	3.0	±SD1.5	0.46
PR (n=15)	3.1	±SD1.4		PR (n=9)	2.9	±SD0.8	
SD (n=0)	-	-		SD (n=2)	4.4	±SD2.1	
PD (n=3)	2.4	±SD0.4		PD (n=16)	2.8	±SD1.2	
**Lymphocytes** count (cells/µL), mean±SD na=6				Lymphocytes count (cells/µL), mean±SD na=4			
CR (n=29)	1.3	±SD0.6	0.96	CR (n=20)	1.2	±SD0.6	0.14
PR (n=14)	1.3	±SD0.6		PR (n=9)	1.0	±SD0.3	
SD (n=0)	-	-		SD (n=2)	2.1	±SD1.3	
PD (n=3)	1.2	±SD0.2		PD (n=16)	1.4	±SD0.6	
**Platelets** median ($x10^3$/µL), mean±SD na=5				Platelets median ($x10^3$/µL), mean±SD na=4			
CR (n=29)	105	±SD50.4	0.13	CR (n=20)	97.1	±SD58	0.73
PR (n=15)	88	±SD50.5		PR (n=9)	93.5	±SD52	
SD (n=0)	-	-		SD (n=2)	51.5	±SD0.2	
PD (n=3)	157	±SD97.0		PD (n=16)	89.5	±SD55	
**NLR**, mean±SD na=1				NLR, mean±SD na=3			
CR (n=32)	2.63	±SD1.5	0.69	CR (n=19)	2.84	±SD1.7	0.38
PR (n=16)	2.45	±SD0.7		PR (n=8)	2.81	±SD0.9	
SD (n=0)	-	-		SD (n=2)	2.24	±SD0.4	
PD (n=3)	2.01	±SD0.4		PD (n=15)	2.14	±SD0.8	
**PLR**, mean±SD na=6				PLR, mean±SD na=4			
CR (n=29)	103	±SD57	0.03	CR (n=19)	97	±SD58	0.73

PR (n=14)	66	±SD24		PR (n=7)	94	±SD52	
SD (n=0)	-	-		SD (n=2)	52	±SD0.2	
PD (n=3)	143	±SD119		PD (n=15)	89	±SD55	
**LCRR**, mean±SD na=8				LCRR, mean±SD na=7			
CR (n=26)	2.5	±SD0.5	0.70	CR (n=18)	3.2	±SD6.4	0.45
PR (n=15)	1.4	±SD0.2		PR (n=7)	0.4	±SD0.3	
SD (n=0)	-	-		SD (n=2)	3.8	±SD1.2	
PD (n=3)	0.8	±SD0.1		PD (n=13)	1.4	±SD2.4	
**Focal nodular HCC** (one nodule), N (%) na=5				Focal nodular HCC (one nodule), N (%) na=5			
CR (n=30)	14	46.7%	0.82	CR (n=19)	13	68.4%	0.10
PR (n=14)	7	50.0%		PR (n=7)	2	28.6%	
SD (n=0)	-	-		SD (n=2)	1	50.0%	
PD (n=3)	1	66.7%		PD (n=14)	4	28.6%	
**CHILD-PLUG** B, N (%) na=10				CHILD-PLUG, N (%) na=9			
CR (n=27)	3	11%	0.56	CR (n=16)	2	13%	0.75
PR (n=12)	2	17%		PR (n=6)	0	-	
SD (n=0)	0	-		SD (n=2)	0	-	
PD (n=3)	1	33%		PD (n=14)	2	15%	
**MELD**, mean±SD na=1				Mayo End Stage Liver Disease (MELD), mean±SD			
CR (n=32)	9	±SD2	0.59	CR (n=20)	10	±SD3	0.62
PR (n=16)	10	±SD2		PR (n=9)	10	±SD2	
SD (n=0)	-	-		SD (n=2)	10	±SD4	
PD (n=3)	10	±SD1		PD (n=16)	10	±SD2	

CR=complete response, PR=partial response, SD=stable disease, PD=progressive disease, NLR=ratios between absolute neutrophils count and absolute lymphocytes count, PLR= platelets and lymphocytes ratio, LCRR=lymphocytes and C-reactive protein ratio;MELD=Mayo End Stage Liver Disease; BCLC= Barcelona Clinic Liver Cancer. na= data not available. Differences among groups was determined by ANOVA and Chi-square test.

Supplementary Table 2. Comparison of clinical and categorical variables for both complete response (CR) vs non-complete response (non-CR) and overall responders (CR+PR) vs non-responders (SD+PD) at 1 and 6 months.

A. Complete Response (CR) vs non-CR

Variable	1-month CR	Non-CR	P	6-month CR	Non-CR	P
Male sex	21/30 (70.0%)	16/19 (84.2%)	0.37	17/22 (77.3%)	20/27 (74.1%)	1.00
Smokers or ex smokers	9/25 (36.0%)	10/12 (83.3%)	0.01*	6/19 (31.6%)	14/19 (73.7%)	0.02*
Non-exotoxic etiology #	14/25 (53.8%)	12 /14 (85.7%)	0.08	9/20 (45.0%)	17/21 (81.0%)	0.02*
Diabetes	8/27 (29.6%)	3/14 (21.4%)	0.72	5/20 (25.0%)	6/21 (28.6%)	1.00
Tumor size, mm	30.0 (19.0-47.5)	30.5 (17.5-81.5)	0.73	25.5 (17.5-43.5)	33.0 (18-77.0)	0.25
Portal vein thrombosis	3/27 (11.1%)	1/14 (7.1%)	1.00	3/20 (15.0%)	1/21 (4.8%)	0.34
BCLC stage 0A vs B	17/28 (60.7%)	10/16 (64.7%)	1.00	8/21 (38.1%)	6/23 (26.1%)	0.44
Creatinine, mg/dL	0.92 ± 0.3	0.83 ± 0.2	0.82	1.04 ± 0.4	0.83 ± 0.2	0.08

B. Overall Responders (CR+PR) vs Non-responders (SD+PD)

Variable	CR+PR (1 mo) n/N (%)	SD+PD (1 mo) n/N (%)	p	CR+PR (6 mo) n/N (%)	SD+PD (6 mo) n/N (%)	p
Male sex	34/45 (75.6%)	3/4 (75.0%)	1.00	23/31 (74.2%)	14/18 (77.8%)	1.00
Overweight (BMI≥25)	22/45 (48.9%)	2/4 (50.0%)	1.00	17/31 (54.8%)	7/18 (38.9%)	0.38
Diabetes	9/38 (23.7%)	2/3 (66.7%)	0.17	6/25 (24.0%)	5/16 (31.3%)	0.72
Hepatitis B/C	19/38 (50.0%)	1/3 (33.3%)	1.00	13/25 (52.0%)	7/16 (43.8%)	0.75
Non-exotoxic etiology #	24/38 (63.2%)	2/3 (66.7%)	1.00	14/25 (56.0%)	12/16 (75.0%)	0.32
Smokers or ex smokers	19/37 (51.4.5%)	1/1 (100.0%)	1.00	10/24 (41.7%)	10/14 (71.4%)	0.10
Alcohol consumption (current or former)	26/37 (70.3%)	2/3 (66.7%)	1.00	16/24 (66.7%)	12/16 (75.0%)	0.73
Focal nodular HCC (one nodule)	16/38 (42.1%)	3/3 (33.3%)	0.09	13/25 (52.0%)	10/16 (62.5%)	0.52
Portal vein thrombosis	4/38 (10.5%)	0/3 (0.0%)	1.00	4/25 (16.0%)	0/16 (0.0%)	0.14
Child-Plug (0A vs B)	32/36 (88.9%)	2/3 (66.7%)	0.34	19/23 (82.6%)	15/16 (6.9%)	0.63
BCLC stage 0A vs B	26/41 (36.6%)	1/3 (33.3%)	0.55	20/28 (71.4%)	7/16 (56.3%)	0.11

Data are expressed as number of positive cases / total patients (%).
The number of N patients (tot data) may differ across variables and between columns due to missing data
*#, metabolic or alcohol consumption; * p-value ≤0.05 (Fisher exact test)*

Supplementary Table 3. Comparison of clinical and biochemical continuous variables for both complete response (CR) vs non-complete response (non-CR) and overall responders (CR+PR) vs non-responders (SD+PD) over time

A. Complete Response (CR) vs non-CR

Variable	CR (1 mo) n/N median [IQR]	Non-CR (1 mo) n/N median [IQR]	p	CR (1 mo) n/N median [IQR]	Non-CR (1 mo) n/N median [IQR]	P
Age, years	29/48 65.12 [61.57–73.64]	19/48 68.42 [63.13–73.57]	0.66	19/44 69.50 [64.31–75.28]	25/44 64.11 [61.57–73.04]	0.18
Overweight, N (%)	29/48 25.30 [23.10–29.59]	19/48 25.70 [23.15–29.52]	0.73	19/44 24.00 [22.75–26.75]	25/44 26.40 [23.40–30.30]	0.18
Albumin (g/L)	24/41 39.30 [37.17–42.00]	17/41 37.90 [36.10–41.00]	0.53	16/38 39.35 [38.22–42.80]	22/38 37.70 [35.20–40.85]	0.05
Total bilirubin (mg/dL)	29/47 0.99 [0.81–1.35]	18/47 1.28 [0.98–1.76]	0.12	19/44 0.97 [0.81–1.18]	25/44 1.21 [0.89–1.66]	0.18
Alfa-feto protein (ng/ml)	29/47 5.70 [3.90–12.60]	18/47 9.40 [7.80–15.25]	0.18	19/44 5.40 [4.55–9.40]	25/44 8.90 [5.70–19.20]	0.11
Creatinine (mg/dL)	29/47 0.84 [0.72–1.08]	18/47 0.83 [0.77–0.96]	0.99	19/44 1.00 [0.79–1.15]	25/44 0.81 [0.75–0.91]	0.06
Hemoglobin (g/dL)	29/47 13.10 [11.70–14.70]	18/47 14.10 [13.55–14.67]	0.18	19/44 14.00 [12.30–15.80]	25/44 13.80 [12.90–14.60]	0.74
C reactive protein (mg/dL),	25/43 1.59 [0.63–3.90]	18/43 3.34 [1.22–5.08]	0.19	18/39 0.98 [0.62–3.14]	21/39 3.05 [1.25–4.92]	0.07
Platelets ($x10^3$/µL),	29/47 90.00 [68.00–143.00]	18/47 75.00 [60.75–138.50]	0.46	19/44 90.00 [65.50–134.00]	25/44 74.00 [63.00–142.00]	0.91
White blood cell (cells/µL)	29/47 4.46 [3.15–6.36]	18/47 4.54 [3.74–5.64]	0.78	19/44 4.82 [3.14–6.43]	25/44 4.46 [3.67–5.44]	0.87
Lymphocytes (cells/µL),	29/46 1.11 [0.77–1.74]	17/46 1.16 [0.90–1.36]	0.66	19/43 1.12 [0.73–1.66]	24/43 1.10 [0.89–1.37]	0.79
Neutrophils (cells/µL)	29/47 2.51 [1.82–3.64]	18/47 2.83 [2.15–3.13]	0.42	19/44 2.73 [1.83–3.77]	25/44 2.58 [2.06–2.95]	0.89
Coagulation (INR index)	29/47 1.15 [1.08–1.19]	18/47 1.20 [1.11–1.29]	0.12	19/44 1.15 [1.08–1.18]	25/44 1.19 [1.11–1.26]	0.15
LCRR	25/42 6860.00 [2700.00–18060.00]	17/42 4040.00 [1860.00–9583.00]	0.57	18/39 9145.00 [2842.50–21052.50]	21/39 3330.00 [1830.00–9583.00]	0.15
NLR	29/47 2.12 [1.47–3.90]	18/47 2.29 [1.90–2.67]	0.71	19/44 2.17 [1.75–3.97]	25/44 2.28 [1.59–2.73]	0.46
PLR	29/46 90.00 [62.38–126.45]	17/46 72.05 [51.70–88.04]	0.15	19/43 79.56 [54.09–131.25]	24/43 74.35 [64.15–92.58]	0.57
Tumor size, mm	27/43 30.00 [19.00–43.50]	16/43 30.50 [18.25–79.25]	0.59	18/39 24.00 [16.75–35.75]	21/39 34.00 [19.00–65.00]	0.22
MELD	29/47 9.00 [8.00–11.00]	18/47 9.00 [8.25–12.00]	0.34	19/44 9.00 [8.00–11.50]	25/44 9.00 [8.00–11.00]	0.75
PMI_2	27/44 44.24 [40.73–49.54]	17/44 45.76 [44.68–48.10]	0.31	19/44 43.94 [39.59–46.81]	25/44 46.84 [41.67–48.98]	0.05
PMI_post	29/48 44.64 [40.30–48.91]	19/48 48.46 [46.85–50.40]	0.03	19/44 43.13 [39.60–47.70]	25/44 48.46 [46.02–51.94]	0.01
PMI_pre	29/48 46.02 [43.56–52.20]	19/48 49.95 [48.96–52.48]	0.14	19/44 45.68 [41.96–50.77]	25/44 49.63 [45.88–54.33]	0.03

B. Overall Responders (CR+PR) vs Non-responders (SD+PD)

Variable	Responders (CR+PR, 1 mo) n/N median [IQR]	Non-Responders (SDR+PD, 6 mo) n/N median [IQR]	p	Responders (CR+PR, 1 mo) n/N median [IQR]	Non-Responders (SDR+PD, 6 mo) n/N median [IQR]	p
Age, years	44/48 65.02 [61.45–73.19]	4/48 73.42 [70.74–76.73]	0.07	27/44 65.43 [61.34–73.87]	17/44 65.06 [62.69–73.72]	0.89
Overweight, N (%)	44/48 25.65 [23.17–29.63]	4/48 24.46 [21.45–28.70]	0.70	27/44 24.40 [22.65–28.68]	17/44 26.40 [24.90–29.74]	0.36
Albumin (g/L)	39/41 39.30 [36.65–41.90]	2/41 39.05 [37.58–40.52]	1.00	24/38 39.30 [37.48–41.85]	14/38 38.65 [35.20–41.75]	0.43
Total bilirubin (mg/dL)	44/47 1.02 [0.85–1.57]	3/47 1.81 [1.35–1.82]	0.35	27/44 0.99 [0.85–1.18]	17/44 1.43 [0.89–1.81]	0.11
Alfa-feto protein (ng/ml)	44/47 7.80 [4.73–14.15]	3/47 10.80 [7.25–15.00]	0.79	27/44 6.20 [3.90–9.40]	17/44 9.90 [6.20–23.60]	0.06
Creatinine (mg/dL)	44/47 0.84 [0.75–1.06]	3/47 0.81 [0.79–0.86]	0.78	27/44 0.88 [0.76–1.09]	17/44 0.79 [0.76–0.87]	0.10
Hemoglobin (g/dL)	44/47 13.75 [12.07–14.70]	3/47 14.50 [13.75–14.80]	0.50	27/44 13.90 [12.05–14.80]	17/44 13.80 [13.00–14.50]	0.86

C reactive protein (mg/dL),	39/43	2.23 [0.85–4.52]	4/43	4.79 [3.50–12.27]	0.23	25/39	1.59 [0.77–3.90]	14/39	2.64 [1.05–5.08]	0.37
Platelets (x10^3/μL),	44/47	82.00 [63.75–142.25]	3/47	104.00 [101.00–186.50]	0.16	27/44	82.00 [65.50–135.00]	17/44	74.00 [63.00–142.00]	0.80
White blood cell (cells/μL)	44/47	4.54 [3.44–6.17]	3/47	3.94 [3.70–4.47]	0.54	27/44	4.60 [3.14–6.24]	17/44	4.35 [3.67–5.40]	0.92
Lymphocytes (cells/μL),	43/46	1.11 [0.78–1.66]	3/46	1.35 [1.16–1.35]	0.62	26/43	0.93 [0.78–1.42]	17/43	1.16 [0.90–1.56]	0.32
Neutrophils (cells/μL)	44/47	2.75 [1.93–3.57]	3/47	2.21 [2.17–2.58]	0.81	27/44	2.82 [1.83–3.66]	17/44	2.51 [2.06–2.91]	0.83
Coagulation (INR index)	44/47	1.15 [1.11–1.22]	3/47	1.20 [1.11–1.21]	0.88	27/44	1.15 [1.12–1.19]	17/44	1.20 [1.10–1.26]	0.33
LCRR	39/42	6280.00 [1845.00–11230.00]	3/42	2650.00 [2405.00–11552.00]	0.85	25/39	6860.00 [2700.00–10630.00]	14/39	3050.00 [1912.50–18298.00]	0.61
NLR	44/47	2.22 [1.65–3.52]	3/47	2.16 [1.87–2.23]	0.63	27/44	2.30 [1.86–3.74]	17/44	2.16 [1.55–2.50]	0.17
PLR	43/46	76.66 [56.27–104.50]	3/46	77.00 [74.53–178.60]	0.40	26/43	84.14 [55.91–117.63]	17/43	68.58 [64.74–96.10]	0.49
Tumor size, mm	40/43	28.50 [18.50–47.75]	3/43	111.00 [60.50–120.00]	0.34	24/39	25.50 [18.25–42.75]	15/39	33.00 [19.50–77.00]	0.25
MELD	44/47	9.00 [8.00–11.25]	3/47	9.00 [9.00–10.00]	0.79	27/44	9.00 [8.00–11.00]	17/44	11.00 [9.00–12.00]	0.34
PMI_2	41/44	45.29 [41.27–48.38]	3/44	45.49 [43.58–47.23]	0.82	27/44	44.62 [40.73–48.53]	17/44	45.49 [41.62–48.10]	0.45
PMI_post	44/48	47.24 [41.87–50.25]	4/48	47.50 [45.66–48.36]	0.90	27/44	45.25 [40.90–48.96]	17/44	48.33 [42.59–52.79]	0.15
PMI_pre	44/48	49.55 [44.26–53.20]	4/48	47.76 [45.83–49.92]	0.74	27/44	47.46 [43.06–51.72]	17/44	49.38 [45.68–53.50]	0.32

Continuous variables were expressed as median (interquartile range, IQR) and compared using Mann–Whitney U test, as most variables were not normally distributed, according to the Shapiro-Wilk test.
